# Electromechanical Impedance Response of a Cracked Timoshenko Beam

**DOI:** 10.3390/s110707285

**Published:** 2011-07-22

**Authors:** Yuxiang Zhang, Fuhou Xu, Jiazhao Chen, Cuiqin Wu, Dongdong Wen

**Affiliations:** 203 Office, Xi’an Research Institute of High-Technology, Xi’an 710025, China; E-Mails: yuxiangz@tom.com (Y.Z.); jazhch@sina.com (J.C.); cuiqin-wu@163.com (C.W.); wdd1949@163.com (D.W.)

**Keywords:** electromechanical impedance, structural health monitoring, PZT, Timoshenko beam

## Abstract

Typically, the Electromechanical Impedance (EMI) technique does not use an analytical model for basic damage identification. However, an accurate model is necessary for getting more information about any damage. In this paper, an EMI model is presented for predicting the electromechanical impedance of a cracked beam structure quantitatively. A coupled system of a cracked Timoshenko beam with a pair of PZT patches bonded on the top and bottom surfaces has been considered, where the bonding layers are assumed as a Kelvin-Voigt material. The shear lag model is introduced to describe the load transfer between the PZT patches and the beam structure. The beam crack is simulated as a massless torsional spring; the dynamic equations of the coupled system are derived, which include the crack information and the inertial forces of both PZT patches and adhesive layers. According to the boundary conditions and continuity conditions, the analytical expression of the admittance of PZT patch is obtained. In the case study, the influences of crack and the inertial forces of PZT patches are analyzed. The results show that: (1) the inertial forces affects significantly in high frequency band; and (2) the use of appropriate frequency range can improve the accuracy of damage identification.

## 1. Introduction

In recent years, the Electromechanical Impedance (EMI) technique has emerged as a promising structural health monitoring (SHM) method. It has been successfully applied to various engineering disciplines, including aerospace and aircraft structures [1–6] and civil structures [7–9]. In this technique, a piezoelectric ceramic lead-zirconate-titanate (PZT) patch is surface bonded to the monitored structure or embedded into a new composite construction and excited by an alternating voltage sweeping signal. Any physical change in the structures will result in a change of the structural mechanical impedance. Due to the electromechanical coupling between the PZT transducers, the EMI signature extracted from PZT transducer is directly related to the mechanical impedance of the monitored structure. Consequently, any structural physical change will induce changes in the EMI signature of the PZT transducer. Hence, for SHM applications, PZT EMI spectra can be compared with a baseline measurement during the service period of the monitored structure. Any change in the spectra is an indication of a change of the structural integrity, which may be caused by the presence of damage.

Although the EMI-based SHM does not typically use an analytical model for basic damage identification, it is necessary to establish a model for more advanced features of SHM, such as damage evaluation and prognosis [10]. Many researchers have investigated the EMI model. As early as the 1990s, Liang *et al.* [11] proposed the first one-dimensional (1D) EMI-type model for a PZT-structure interaction system. In his model the electromechanical admittance (inverse of impedance) of PZT can be expressed analytically by structure impedance. Zhou *et al.* [12] extended the 1D impedance method to model a two-dimensional (2D) PZT element coupled to a 2D monitored structure. On the basis of the concept of “effective impedance”, Bhalla and Soh [13,14] improved Zhou’s model. Yang presented a generic model for predicting the electromechanical impedance of one-dimensional and two-dimensional PZT—structure interaction systems [15]. Based on the concept “sum impedance”, Annamdas and Soh [16,17] proposed a three-dimensional (3D) EMI model. In general, all the models above ignored the adhesive layer between the PZT transducer and the monitored structure. However, many experimental and theoretical analysis results [18–22] have demonstrated that the mechanical interaction between the PZT transducer and the monitored structure occurs through interfacial shear stress. Hence, the adhesive layer has to be considered. Ong *et al.* presented an EMI model which considered the shear lag effect of the bond layer [23]. Suresh Bhalla *et al.* [24] incorporated the shear lag effect into the existing 1D and 2D EMI and obtained an improved model for them. Yan *et al.* [25,26] considered the adhesive layer, and presented an EMI model for Timoshenko beams and Mindlin-Herrmann rods. Although the factor of the adhesive layer was considered, the inertial terms of PZT and adhesive layer produced by the motion along with the monitored structure have not been taken into account in the above models. Pietrzakowski [22] noticed the inertial terms of PZT patches and studied the influence of bonding layer on the beam response, but his method is not suitable for high frequency EMI techniques due to the use of Euler beams.

For some simple pristine structure, the analytical EMI model can be obtained. However, when damages are induced in structures resulting in the possibly inhomogeneity of material properties, it is difficult to derive an analytical formulation. In order to quantitatively identify structural damages, numerical methods and approximate approaches have been adopted. Naidu and Soh [27] and Tseng and Wang [28] obtained the relationships between the EMI signatures and the structural changes by the Finite Element Method (FEM). However, for the purpose of predicting the response accurately in a high frequency range, a very small size element is needed. Therefore the FEM method is time-consuming. Yang [29] and Xu [30] applied the p-Ritz method to establish EMI models for health monitoring of beams, plates and cylindrical shell structures with various boundaries. Their models could calculate the EMI response effectively and accurately below 50 kHz, however, the accuracy of prediction was reduced with the increased frequency.

In order to quantitatively identify the structural damage in a more efficient way, a more accurate EMI model is needed. This study proposes an electromechanical impedance model for health monitoring of beam structures. Different to the existing EMI model, the proposed model not only contains physical parameters of damage and the shear lag effect of the bonding layer, but also includes the transversal inertial forces of PZT patches and adhesive induced by the transversal motion of beam structures.

In this paper, a pair of PZT patches bonded symmetrically onto the top and bottom surfaces of a rectangular beam with a crack was activated out of phase to create a pure bending excitation. Pure extensions in PZT patches were assumed. A shear lag model was applied to describe the behavior of bonding layer which is assumed to be a Kelvin-Voigt material. The PZT-adhesive layer-cracked beam coupled structural system was considered. The coupled system is divided into four sections due to the crack cross-section and the location of PZT patches. The crack is simulated by a massless torsional spring. Taking into account the inertial forces of PZT patches and bonding layer, the boundary problem is formulated by the dynamic equations of the coupled system. According to the boundary conditions and continuity conditions, the solution of the coupled system can be obtained, and then the analytical relationship between PZT admittance (inverse of impedance) and the damage parameters such as location and depth is derived. Finally, numerical results are presented and discussed to validate the proposed theoretical analysis.

## 2. Formulation of the Coupled Structure and Analysis

**Figure 1.**
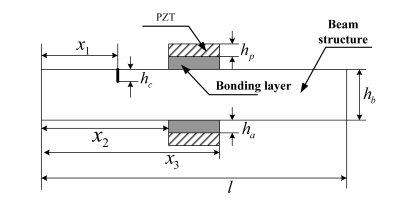
Beam with a pair PZT patches bonded on its surface.

As shown in Figure 1, two identical PZT patches are bonded symmetrically onto the top and bottom surfaces of the Timoshenko beam. Two out-of-phase alternating electrical fields are applied to the PZT patches.

The beam was activated to create a pure bending vibration. A crack with a depth of *h_c_* is located at *x*_1_. For a better analysis, the beam is divided into four parts due to the crack cross-section and the location of PZT patch (Figure 1). The dynamic behavior of each part is governed by different equations. According to boundary conditions and continuity conditions, the dynamic equations of PZT-structure interaction system can be solved. The analysis method used here is similar to the method used by Pietrzakowski [22], but different from that work, the Timoshenko beam theory and the inertial of bonding layers are taken into account in this paper.

### 2.1. Dynamic Equations

**Figure 2.**
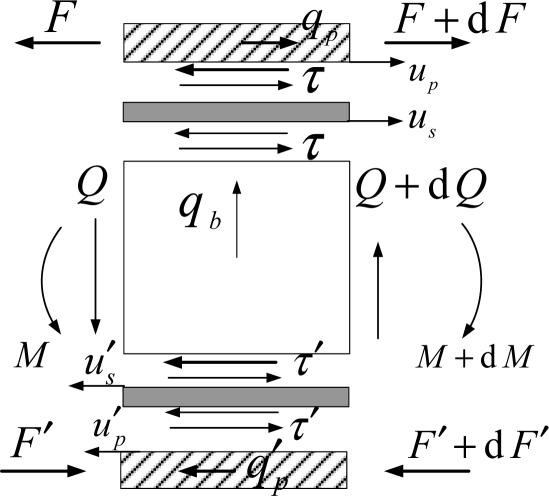
Infinitesimal beam element with piezoelements and bonding layers.

The governing equations for the section with PZT patch (*x*_2_ < *x* < *x*_3_) are derived considering an infinitesimal element shown in Figure 2. The longitudinal motion of the PZT patches is defined by introducing inertial forces. The governing equations can be expressed as:

(1) $$\begin{array}{l} E_{\text{p}}\frac{\partial^{2}u_{\text{p}}}{\partial x^{2}}-\rho_{\text{p}}\frac{\partial^{2}u_{\text{p}}}{\partial t^{2}}-\frac{\tau }{h_{\text{p}}}=0\\ E_{\text{p}}\frac{\partial^{2}u_{\text{p}}^{\prime }}{\partial x^{2}}-\rho_{\text{p}}\frac{\partial^{2}u_{\text{p}}^{\prime }}{\partial t^{2}}-\frac{\tau \prime }{h_{\text{p}}}=0 \end{array}$$

where *E*_p_, *ρ*_p_, *h*_p_, denote Young’s modulus, density and thickness of the two PZT patches, respectively; *u*_p_ and $u_{\text{p}}^{\prime }$ are longitudinal displacement of the upper PZT and the lower PZT, respectively; The shear stresses *τ* and *τ′* transmitted by the piezoelements are determined by the following stress-strain relation:

(2) $$\begin{array}{l} \tau =\frac{G_{a}^{*}(u_{\text{p}}-u_{\text{s}})}{h_{\text{a}}}\\ \tau^{\prime }=\frac{G_{a}^{*}(u_{\text{p}}^{\prime }-u_{\text{s}}^{\prime })}{h_{\text{a}}} \end{array}$$

where *u_s_* and $u_{\text{s}}^{\prime }$ are the upper and lower beam surface longitudinal displacement, respectively; *h*_a_ is the bonding layer thickness; *h*_b_ is the beam thickness; and *w* is the transverse displacement of beam; $G_{a}^{*}$ is the linear function of differential operator which for the Kelvin-Voigt model of bonding layer material can be expressed as:

(3) $$G_{a}^{*}=G_{a}(1+C_{b}\frac{\partial }{\partial t})$$

where *G*_a_ is Kirchhoff’s modulus; *C*_b_ denotes the retardation time.

Because the two PZT patches are identical and are actived by two out-of-phase alternating electrical fields with same amplitude, so, the following relationship can be obtained:

(4) $$\begin{array}{l} \tau^{\prime }=\tau \\ u_{\text{p}}^{\prime }=u_{\text{p}}\\ u_{\text{s}}^{\prime }=u_{\text{s}}=-\frac{h_{b}}{2}\frac{\partial w}{\partial x} \end{array}$$

Considering the dynamic coupling between the piezoelement and the beam, and including the mass of piezoelement and adhesive in the inertial force of intensity *q_b_* (shown in Figure 2), the transverse motion of Timoshenko beam is described. The governing equations of beam are expressed as follows:

(5) $$\frac{\partial M}{\partial x}+\tau h_{\text{b}}b_{\text{b}}-Q+\gamma \rho_{\text{b}}I\frac{\partial^{2}\phi }{\partial t^{2}}=0,\;\frac{\partial Q}{\partial x}-\gamma \rho_{\text{b}}A_{\text{b}}\frac{\partial^{2}w}{\partial t^{2}}=0,\;M=-E_{\text{b}}I\frac{\partial \phi }{\partial x},\;Q=\kappa A_{\text{b}}G_{\text{b}}(\frac{\partial w}{\partial x}-\phi )$$

where *E_b_*, *G_b_* are the Young’s modulus and shear modulus, respectively; *κ* = *π*^2^/12 is the shear correction factor; *I* is the inertia moment; *M*, *Q* are the bending moment, transverse force of beam, respectively; *ϕ* is the rotation angle of beam due to pure bending; *b*_b_, *ρ*_b_, *A*_b_ denote the width, density and cross-section area of beam, respectively; the mass ratio *γ* is determined by following relation:

(6) $$\begin{array}{l} \gamma =\frac{\rho_{\text{b}}A_{\text{b}}+2\rho_{\text{p}}A_{\text{p}}+2\rho_{\text{a}}A_{\text{a}}}{\rho_{\text{b}}\;A_{\text{b}}}\\ A_{\text{b}}=h_{\text{b}}b_{\text{b}},A_{\text{a}}=h_{\text{a}}b_{\text{a}},A_{\text{p}}=h_{\text{p}}b_{\text{p}} \end{array}$$

where *ρ*_a_ is the density of adhesive layer.

From Equations (1–5), the following equations can be obtained:

(7) $$E_{\text{p}}\frac{\partial^{2}u_{\text{p}}}{\partial x^{2}}-\frac{G_{\text{a}}^{*}}{h_{\text{p}}h_{\text{a}}}(u_{\text{p}}+\frac{h_{\text{b}}}{2}\frac{\partial w}{2})=\rho_{\text{p}}\frac{\partial^{2}u_{\text{p}}}{\partial t^{2}}$$

(8) $$-E_{\text{b}}I\frac{\partial^{4}w}{\partial x^{4}}+h_{\text{b}}b_{\text{b}}\frac{G_{\text{a}}^{*}}{h_{\text{a}}}(\frac{\partial u_{\text{p}}}{\partial x}+\frac{h_{\text{p}}}{2}\frac{\partial^{2}w}{\partial x^{2}})-I\gamma \rho_{\text{b}}(\frac{E_{\text{b}}}{\kappa G_{\text{b}}}+1)\frac{\partial^{4}w}{\partial x^{2}\partial t^{2}}-\gamma \rho_{\text{b}}A_{\text{b}}\frac{\partial^{2}w}{\partial t^{2}}-\frac{I\gamma^{2}\rho_{\text{b}}^{2}}{\kappa G_{\text{b}}}\frac{\partial^{4}w}{\partial t^{4}}=0$$

Assuming the high frequency harmonic voltage loaded on the upper and lower PZT patch are V(*x*,*t*) = V̄(*x*)e*^iωt^* and V′(*x*,*t*) = V̄(*x*)e*^i^*^(^*^ωt+π^*^)^: then the steady state response of coupled structural system can be expressed as:

(9) $$u_{\text{p}}(x,t)={\bar{u}}_{\text{p}}(x)e^{\mathrm{i\omega t}},u_{s}(x,t)={\bar{u}}_{s}(x)e^{\mathrm{i\omega t}},\tau (x,t)=\bar{\tau }(x){\text{e}}^{\mathrm{i\omega t}},w(x,t)=\bar{w}(x){\text{e}}^{\mathrm{i\omega t}}$$

From Equation (8) and Equation (9), we can obtain:

(10) $${\bar{ɛ}}_{\text{p}}=\frac{\partial {\bar{u}}_{\text{p}}}{\partial x}=\frac{E_{\text{b}}Ih_{\text{a}}}{h_{\text{b}}b_{\text{b}}{\bar{G}}_{\text{a}}}\frac{\partial^{4}\bar{w}}{\partial x^{4}}-\frac{h_{\text{b}}}{2}\frac{\partial^{2}\bar{w}}{\partial x^{2}}+\frac{Ih_{\text{a}}\gamma \rho_{\text{b}}\omega^{2}}{h_{\text{b}}b_{\text{b}}{\bar{G}}_{\text{a}}}(\frac{E_{\text{b}}}{\kappa G_{\text{b}}}+1)\frac{\partial^{2}\bar{w}}{\partial x^{2}}+(\frac{Ih_{\text{a}}\gamma^{2}\rho_{\text{b}}^{2}\omega^{4}}{h_{\text{b}}b_{\text{b}}{\bar{G}}_{\text{a}}\kappa G_{\text{b}}}-\frac{h_{\text{a}}\gamma \rho_{\text{b}}A_{\text{b}}\omega^{2}}{h_{\text{b}}b_{\text{b}}{\bar{G}}_{\text{a}}})\bar{w}$$

where, *Ḡ*_a_ = *G*_a_ (1 + *iC*_b_*ω*).

Substituting Equation (10) into Equation (7), the following equation can be obtained:

(11) $$\frac{{\text{d}}^{6}\bar{w}}{\text{d}x^{6}}+k_{1}\frac{{\text{d}}^{4}\bar{w}}{\text{d}x^{4}}+k_{2}\frac{{\text{d}}^{2}\bar{w}}{\text{d}x^{2}}+k_{3}\bar{w}=0$$

where:

$$\begin{array}{c} k_{1}=-(\frac{h_{\text{b}}^{2}b_{\text{b}}{\bar{G}}_{\text{a}}}{2E_{\text{b}}Ih_{\text{a}}}+\frac{{\bar{G}}_{\text{a}}}{h_{\text{p}}h_{\text{a}}E_{\text{p}}}-\frac{\rho_{\text{p}}\omega^{2}}{E_{\text{p}}}-\frac{\gamma \rho_{\text{b}}\omega^{2}}{\kappa G_{\text{b}}}-\frac{\gamma \rho_{\text{b}}\omega^{2}}{E_{\text{b}}})\\ k_{2}=\frac{\omega^{4}\rho_{\text{p}}\gamma \rho_{\text{b}}}{E_{\text{p}}E_{\text{b}}}+\frac{\omega^{4}\gamma^{2}\rho_{\text{b}}^{2}}{\kappa G_{\text{b}}E_{\text{b}}}-\frac{{\bar{G}}_{\text{a}}\gamma \rho_{\text{b}}\omega^{2}}{E_{\text{p}}h_{\text{a}}E_{\text{b}}h_{\text{p}}}-\frac{{\bar{G}}_{\text{a}}\gamma \rho_{\text{b}}\omega^{2}}{E_{\text{p}}h_{\text{a}}h_{\text{p}}\kappa G_{\text{b}}}-\frac{\omega^{2}\gamma \rho_{\text{b}}A_{\text{b}}}{IE_{\text{b}}}+\frac{\omega^{4}\gamma \rho_{\text{b}}\rho_{\text{b}}}{\kappa G_{\text{b}}E_{\text{p}}}-\frac{h_{\text{b}}^{2}b_{\text{b}}{\bar{G}}_{\text{a}}\rho_{\text{p}}\omega^{2}}{2E_{\text{p}}h_{\text{a}}E_{\text{b}}I}\\ k_{3}=-(\frac{{\bar{G}}_{\text{a}}\gamma^{2}\rho_{\text{b}}^{2}\omega^{4}}{h_{p}h_{\text{a}}E_{\text{p}}E_{\text{b}}\kappa G_{\text{b}}}-\frac{\rho_{\text{p}}\gamma^{2}\rho_{\text{b}}^{2}\omega^{6}}{E_{\text{p}}E_{\text{b}}\kappa G_{\text{b}}}-\frac{{\bar{G}}_{\text{a}}\gamma \rho_{\text{b}}A_{\text{b}}\omega^{2}}{h_{\text{p}}h_{\text{a}}E_{\text{p}}E_{\text{b}}I}+\frac{\gamma \rho_{\text{b}}A_{\text{b}}\omega^{4}}{E_{\text{p}}E_{\text{b}}I}) \end{array}$$

The solution of Equation (11) can be expressed as:

(12) $$\bar{w}(x)=c_{1}{\text{e}}^{-\beta_{1}x}+c_{2}{\text{e}}^{\beta_{1}x}+c_{3}{\text{e}}^{-\beta_{2}x}+c_{4}{\text{e}}^{\beta_{2}x}+c_{5}{\text{e}}^{-\beta_{3}x}+c_{6}{\text{e}}^{\beta_{3}x},x\in [x_{2}^{+},x_{3}^{-}]$$

where, *c_i_* (*i* = 1,⋯,6) are the undetermined constant coefficients, ± *β_i_* (*i* = 1,2,3) are the characteristic roots of the following equation:

(13) $$\beta^{6}+k_{1}\beta^{4}+k_{2}\beta^{2}+k_{3}=0$$

Substituting the Equation (12), Equation (9) into Equation (10), we can obtain:

(14) $${\bar{ɛ}}_{\text{p}}=g_{1}c_{1}\;{\text{e}}^{-\beta_{1}x}+g_{1}c_{2}\;{\text{e}}^{\beta_{1}x}+g_{2}c_{3}\;{\text{e}}^{-\beta_{2}x}+g_{2}c_{4}\;{\text{e}}^{\beta_{2}x}+g_{3}c_{5}\;{\text{e}}^{-\beta_{3}x}+g_{3}c_{6}\;{\text{e}}^{\beta_{3}x}$$

where

$$g_{i}=\frac{E_{\text{b}}Ih_{\text{a}}}{h_{\text{b}}b_{\text{b}}{\bar{G}}_{\text{a}}}\beta_{i}^{4}-\frac{h_{\text{b}}}{2}\beta_{i}^{2}+\frac{Ih_{\text{a}}\gamma \rho_{\text{b}}\omega^{2}}{h_{\text{b}}b_{\text{b}}{\bar{G}}_{\text{a}}}(\frac{E_{\text{b}}}{\kappa G_{\text{b}}}+1)\beta_{i}^{2}+(\frac{Ih_{\text{a}}\gamma^{2}\rho_{\text{b}}^{2}\omega^{4}}{h_{\text{b}}b_{\text{b}}{\bar{G}}_{a}\kappa G_{\text{b}}}-\frac{h_{\text{a}}\gamma \rho_{\text{b}}A_{\text{b}}\omega^{2}}{h_{\text{b}}b_{\text{b}}{\bar{G}}_{\text{a}}}),(i=1,2,3)$$

Substituting the Equation (12), Equation (9) into Equation (7), we can obtain:

(15) $${\bar{u}}_{\text{p}}=\frac{g_{1}}{-\beta_{1}}c_{1}\;{\text{e}}^{-\beta_{1}x}+\frac{g_{1}}{\beta_{1}}c_{2}\;{\text{e}}^{\beta_{1}x}+\frac{g_{2}}{-\beta_{2}}c_{3}\;{\text{e}}^{-\beta_{2}x}+\frac{g_{2}}{\beta_{2}}c_{4}\;{\text{e}}^{\beta_{2}x}+\frac{g_{3}}{-\beta_{3}}c_{5}\;{\text{e}}^{-\beta_{3}x}+\frac{g_{3}}{\beta_{3}}c_{6}\;{\text{e}}^{\beta_{3}x}$$

By substituting the Equations (2), (11), (15) into Equation (5), respectively, the bending moment *M,* shear stress *Q* and rotational angle *ϕ* can be obtained. In order to solve the unknown constants *c_i_* (i = 1,....,6),the boundary conditions and the continuity conditions are required, so, the analytical expression of other beam section is also needed. The basic equations of a uniform beam section without bonded PZT patches are:

(16) $$\frac{\partial M}{\partial x}-Q+\rho_{\text{b}}I\frac{\partial^{2}\phi }{\partial t^{2}}=0,\frac{\partial Q}{\partial x}-\rho_{\text{b}}A_{\text{b}}\frac{\partial^{2}w}{\partial t^{2}}=0,M=-E_{\text{b}}I\frac{\partial \phi }{\partial x},Q=\kappa A_{\text{b}}G_{\text{b}}(\frac{\partial w}{\partial x}-\phi )$$

From Equation (16), the following relations can be obtained:

(17) $$E_{\text{b}}I\frac{{\text{d}}^{4}\bar{w}}{\text{d}x^{4}}+(\frac{E_{\text{b}}}{\kappa G_{\text{b}}}+1)I\rho_{\text{b}}\omega^{2}\frac{{\text{d}}^{2}\bar{w}}{\text{d}x^{2}}-(\rho_{\text{b}}A_{\text{b}}\omega^{2}-\frac{\rho_{\text{b}}^{2}I\omega^{4}}{\kappa G_{\text{b}}})\bar{w}=0$$

The steady state solution of the Equation (17) can be expressed as:

(18) $$\begin{array}{l} \bar{w}(x)=c_{7}\;{\text{e}}^{\frac{1}{2}k_{4}x}+c_{8}\;{\text{e}}^{\frac{1}{2}k_{4}x}+c_{9}\;{\text{e}}^{\frac{1}{2}k_{5}x}+c_{10}\;{\text{e}}^{\frac{1}{2}k_{5}x},x\in [0,x_{1}^{-}]\\ \bar{w}(x)=c_{11}\;{\text{e}}^{\frac{1}{2}k_{4}x}+c_{12}\;{\text{e}}^{\frac{1}{2}k_{4}x}+c_{13}\;{\text{e}}^{\frac{1}{2}k_{5}x}+c_{14}\;{\text{e}}^{\frac{1}{2}k_{5}x},x\in [x_{1}^{+},x_{2}^{-}]\\ \bar{w}(x)=c_{15}\;{\text{e}}^{\frac{1}{2}k_{4}x}+c_{16}\;{\text{e}}^{\frac{1}{2}k_{4}x}+c_{17}\;{\text{e}}^{\frac{1}{2}k_{5}x}+c_{18}\;{\text{e}}^{\frac{1}{2}k_{5}x},x\in [x_{3}^{+},l] \end{array}$$

where, *c_i_* (*i* = 7,…,18) are the undetermined constant coefficients; and *k*_4_, *k*_5_ can be expressed as:

$$\begin{array}{l} k_{4}={\left(-2k_{6}-2\sqrt{k_{6}^{2}-4k_{7}}\right)}^{1/2},k_{5}={\left(-2k_{6}+2\sqrt{k_{6}^{2}-4k_{7}}\right)}^{1/2}\\ k_{6}=(\frac{1}{\kappa G_{\text{b}}}+\frac{1}{E_{\text{b}}})\rho_{\text{b}}\omega^{2},k_{7}=-(\frac{\rho_{\text{b}}A_{\text{b}}\omega^{2}}{E_{\text{b}}I}-\frac{\rho_{\text{b}}^{2}\omega^{4}}{E_{\text{b}}\kappa G_{\text{b}}}) \end{array}$$

**Figure 3.**
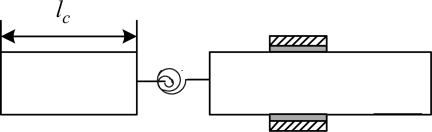
The equivalent model of the cracked beam.

Substituting the Equation (18) into Equation (16), we can obtain the bending moment *M*, shear stress *Q* and rotational angle *ϕ* of the beam without bonded PZT patches. In this study, the crack is assumed to be a fully open crack, and the depth of crack is relative small. Therefore, the crack on the beam can be modeled by a weightless rotational spring [31] as shown in Figure 3. The softness of the spring *θ* is a function of the beam thickness and the depth of crack [31,32] and can be expressed as:

(19) $$\begin{array}{ll} \theta & =6\pi (1-\mu^{2})h_{\text{b}}(0.6272\xi^{2}-0.4533\xi^{3}+4.5948\xi^{4}-9.9736\xi^{5}\\ & +20.2948\xi^{6}-33.0351\xi^{7}+47.1063\xi^{8}-40.7556\xi^{9}+19.6\xi^{10}) \end{array}$$

where *ξ* = *h_c_/h_b_*; *h_c_* is the depth of crack, and *μ* is the Poisson’s ratio of the beam.

Considering the continuity of transverse placement, bending moment, shear stress and discontinuity of slope at the crack, we can obtain the following equation:

(20) $$w^{+}=w^{-},M^{+}=M^{-},Q^{+}=Q^{-},\phi^{+}=\phi^{-}+\theta \frac{\partial \phi^{-}}{\partial x}$$

where, “+” denotes the right side of crack; “−” denotes the left side of crack.

### 2.2. Steady-State Solution

There are 18 undetermined constant coefficients *c_i_* (*i* = 1,⋯,18) in the steady analytical solution of coupled system. The undetermined coefficients can be determined through boundary condition and continuity condition. For the classical end of beam, one has the following equations:

hinged end

(21) w=0,M=0

clamped end

(22) w=0,ϕ=0

free end

(23) M=0,Q=0

For the cantilever beam, the boundary conditions are expressed as:

(24) $$\bar{w}(0)=\bar{\phi }(0)=0$$

(25) $$\bar{M}(0)=\bar{Q}(0)=0$$

The boundary conditions of PZT patch can be expressed as:

(26) $${\bar{ɛ}}_{\text{p}}(x_{2})=d_{31}{\bar{E}}_{3}=\frac{d_{31}\bar{V}}{h_{\text{p}}},{\bar{ɛ}}_{\text{p}}(x_{3})=d_{31}{\bar{E}}_{3}=\frac{d_{31}\bar{V}}{h_{\text{p}}}$$

According to the continuity of beam deflection, slope, bending moment and shear stress at the borders of the sections at *x* = *x*_1_, *x* = *x*_2_, *x* = *x*_3_ we can obtain the following equations:

(27) $$\bar{\phi }(x_{1}^{-})=\bar{\phi }(x_{1}^{+})+\theta (-{\frac{\text{d}\bar{\phi }}{\text{d}x}|}_{x=x_{1}^{-}}),\;\bar{\phi }(x_{2}^{-})=\bar{\phi }(x_{2}^{+}),\;\bar{\phi }(x_{3}^{-})=\bar{\phi }(x_{3}^{+}),$$

(28) $$\bar{M}(x_{1}^{-})=\bar{M}(x_{1}^{+}),\;\bar{M}(x_{2}^{-})=\bar{M}(x_{2}^{+}),\;\bar{M}(x_{3}^{-})=\bar{M}(x_{3}^{+}),$$

(29) $$\bar{Q}(x_{1}^{-})=\bar{Q}(x_{1}^{+}),\;\bar{Q}(x_{2}^{-})=\bar{Q}(x_{2}^{+}),\;\bar{Q}(x_{3}^{-})=\bar{Q}(x_{3}^{+})$$

(30) $$\bar{w}(x_{1}^{-})=\bar{w}(x_{1}^{+}),\;\bar{w}(x_{2}^{-})=\bar{w}(x_{2}^{+}),\;\bar{w}(x_{3}^{-})=\bar{w}(x_{3}^{+})$$

The 18 unknown coefficients *c_i_* = (*i* = 1,⋯,18) are obtained from the system of algebraic equations determined by the boundary and continuity conditions [Equations (24–30)].

## 3. Electromechanical Signatures

Consider a pure extension of PZT patches, and then the corresponding constitutive equations of PZT patches can be expressed as [33]:

(31) $$\begin{array}{l} {ɛ}_{1}=\sigma_{1}/{\bar{E}}_{p}+d_{31}E_{3}\\ D_{3}=d_{31}\sigma_{1}+{\bar{ɛ}}_{33}^{T}E_{3} \end{array}$$

where *ε*_1_, *σ*_1_ are the strain and stress along *x* direction, respectively; *D*_3_, *E*_3_ are the electric flux density and electric field intensity along height direction, respectively; *Ē_p_* = *E_p_* (1 + *jη*) is the complex Young’s modulus of the PZT material at zero electric field with *η* denoting the mechanical loss factor; ${\bar{ɛ}}_{33}^{T}={ɛ}_{33}^{T}(1-j\delta )$ is the complex dielectric constant at zero stress with δ denoting the dielectric loss factor of PZT patch.

From Equation (31), we can obtain the following equation:

(32) $$D_{3}=d_{31}({ɛ}_{1}-d_{31}E_{3}){\bar{E}}_{p}+{\bar{ɛ}}_{33}^{T}E_{3}=d_{31}{\bar{E}}_{p}{ɛ}_{1}+({\bar{ɛ}}_{33}^{T}-d_{31}^{2}{\bar{E}}_{p})E_{3}$$

The electric current passing through the upper PZT patch can be determined from the electric displacement as:

(33) $$\begin{array}{lll} I_{\text{p}} & = & i\omega \int_{0}^{b_{\text{a}}}\int_{x_{2}}^{x_{3}}D_{3}dxdy\\ & = & i\omega b_{\text{a}}d_{31}{\bar{E}}_{\text{p}}[u_{\text{p}}(x_{3})-u_{\text{p}}(x_{2})]+i\omega E_{3}b_{\text{a}}(x_{3}-x_{2})({\bar{ɛ}}_{33}^{T}-d_{31}^{2}{\bar{E}}_{\text{p}}) \end{array}$$

where *I_p_* is the electric current passing through PZT patch.

Electric admittance of the upper PZT patch can be determined as:

(34) $$\begin{array}{lll} Y & = & \frac{I_{p}}{V}=i\omega \frac{b_{\text{a}}d_{31}{\bar{E}}_{\text{p}}({\bar{u}}_{\text{p}}(x_{3})-{\bar{u}}_{\text{p}}(x_{2}))}{\bar{V}}+i\omega \frac{\left({\bar{ɛ}}_{33}^{T}-d_{31}^{2}{\bar{E}}_{\text{p}})b_{\text{a}}(x_{3}-x_{2}\right)}{h_{p}}\\ & = & i\omega \frac{b_{\text{a}}d_{31}{\bar{E}}_{\text{p}}({\bar{u}}_{\text{p}}(x_{3})-{\bar{u}}_{\text{p}}(x_{2}))}{\bar{V}}+i\omega \frac{({\bar{ɛ}}_{33}^{T}-d_{31}^{2}{\bar{E}}_{\text{p}})b_{\text{a}}l_{p}}{h_{\text{p}}}\\ & = & i\omega \frac{b_{\text{a}}d_{31}{\bar{E}}_{\text{p}}}{\bar{V}}\sum_{i=1}^{3}\frac{g_{i}}{\beta_{i}}[({\text{e}}^{\beta_{i}x_{3}}-{\text{e}}^{\beta_{i}x_{2}})c_{2\times i}-({\text{e}}^{-\beta_{i}x_{3}}-{\text{e}}^{-\beta_{i}x_{2}})c_{i}]+i\omega \frac{({\bar{ɛ}}_{33}^{T}-d_{31}^{2}{\bar{E}}_{\text{p}})b_{\text{a}}l_{\text{p}}}{h_{p}} \end{array}$$

where *Y* is the electric admittance of PZT patch.

For a piezoelectric system without damage on the PZT patches, the parameters of the PZT patches can be regarded as constants. Hence, the change of admittance of PZT patch is only determined by the first term on the right side of Equation (34). From Equation (34), we can see that any changes in beam structure will lead to a change of the admittance signature of the PZT patch.

## 4. Numerical Examples

In this paper, a cantilever beam with a pair of PZT patches bonded symmetrically onto its top and bottom surfaces is studied. The PZT patches are located at *x*_2_ = 200 mm from the left side of beam (Figure 1). Geometric parameters and material constants of the beam, PZT patches and adhesive layer are listed in Tables 1–3, respectively.

**Figure 4.**
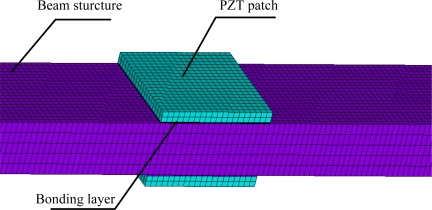
FE model of beam and PZT patches in Ansys.

By considering the inertia terms of PZT patches and bonding layers caused by their motion with the beam, the values of *γ* against the beam thickness *h*_b_ can be calculated and the result is listed in Table 4. If the inertia terms are not taken into account, then *γ* = 1. In order to study the effect of inertia, the differences of the admittances of PZT under two scenarios (considering/without considering the inertia effect) are compared. To verify the accuracy and the reliability of the proposed analytical model, the results are compared to the simulation results obtained by FEA (finite element analysis), which can predict well the experimental results [34]. Yang *et al.* developed a multi-physics simulation method of EMI modeling, which can effect direct acquisition of PZT electrical admittance [35]. Yang’s method was also used in this work. The test specimen is numerically modeled in the ANSYS 10.0 workspace as illustrated in Figure 4. The constitutive data, in accordance to PZT-5A, are assigned to the PZT patch as given in Table 5. The properties of beam and bonding layer are listed in Tables 1 and 3, respectively. The PZT patches are modeled with Solid 5 elements and the bonding layer and beam with Solid 45 elements. The sizes of the elements are less than 1.0 mm. An alternating (sinusoidal) voltage of 1 V was applied across the PZT patch for excitation. The detailed process can be found in the references [34] and [35].

**Figure 5.**
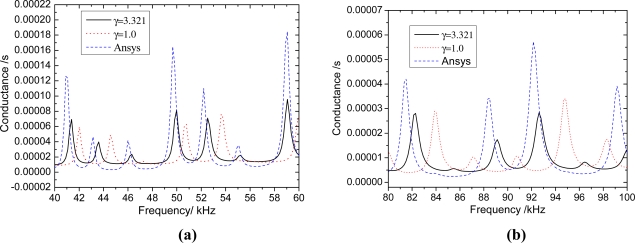
The influence of inertia terms of PZT patches and bonding layers when *h*_b_ = 2.5 mm. **(a)** 40 kHz–60 kHz; **(b)** 80 kHz–100 kHz.

**Figure 6.**
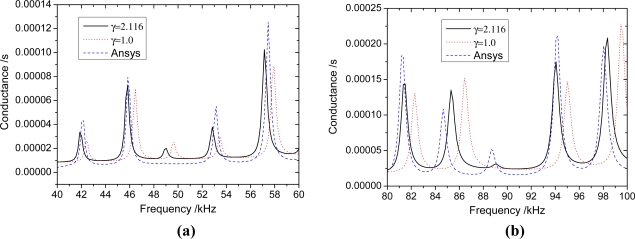
The influence of inertia terms of PZT patches and bonding layers when *h*_b_ = 5 mm. **(a)** 40 kHz–60 kHz; **(b)** 80 kHz–100 kHz.

**Figure 7.**
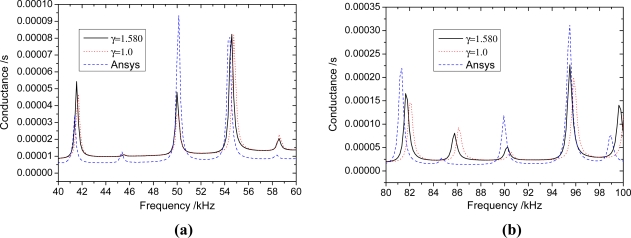
The influence of inertia terms of PZT patches and bonding layers when *h*_b_ = 10 mm. **(a)** 40 kHz–60 kHz; **(b)** 80 kHz–100 kHz.

The results shown in Figures 5–7 suggest that the proposed analytical model provides reasonable predictions of the the FEA results, as the major resonance peaks are well predicted. For any beam thickness *h*_b_, the corresponding frequencies to the peaks of admittance curves considering the inertia terms are smaller than those without considering the inertia terms; and the difference between the curves is increased with the increasing frequency. When *h*_b_ = 10 mm (Figure 7), the difference between curves of *γ* = 1.58 and *γ* = 1.0 is small, however, it is increased with the increasing frequency, and the difference of peak frequency is about 0.45 kHz near 96 kHz. From Figure 7, It can be seen that the inertia effect cannot be ignored if the frequency is high (>80 kHz). When *h*_b_ = 5 mm (Figure 6), the difference between curves of *γ* = 2.116 and *γ* = 1.0 is more obvious than that of *h*_b_ = 10 mm, and the difference of peak frequency is about 1.5 kHz near 98 kHz. From Figure 6, it can be seen that the influence of inertia can’t be ignored when the frequency is higher than 40 kHz. When *h*_b_ = 2.5 mm (Figure 5), the curves of *γ* = 3.321 have a distinct difference with the curve of *γ* = 1.0. The difference of peak frequency between *γ* = 3.321 and *γ* = 1.0 near 41 kHz is about 0.73 kHz.

As shown in Figures 5–7, when the mass ratio *γ* is large, the inertia forces of PZT patches and adhesive layers can influence the admittance signature greatly, even in the low frequency range, and the inertia term must be considered; when the mass ratio *γ* is small, the inertial term also need to be considered in the high frequency range. Because the EMI technique uses high-frequency alternating current, the proposed EMI model which takes inertial term into account will predict more accurate results.

**Figure 8.**
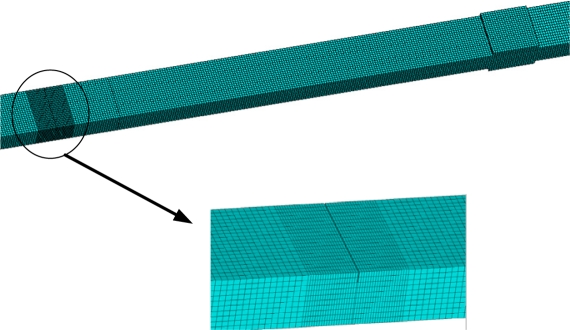
FE model of beam with a crack in Ansys.

**Figure 9.**
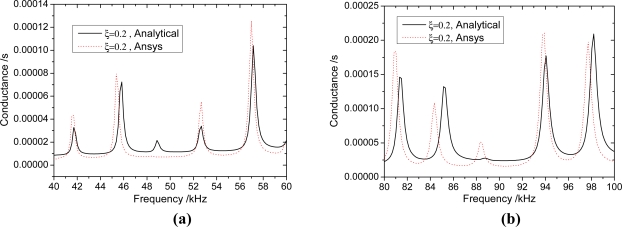
Comparison of admittance signatures against frequency plot between the ANSYS simulation and the analytical results. **(a)** 40 kHz–60 kHz; **(b)** 80 kHz–100 kHz.

**Figure 10.**
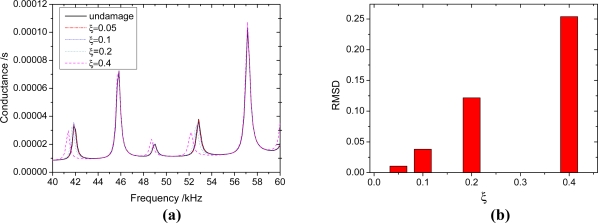
The influence of different extent crack damage in 40 kHz–60 kHz. **(a)** the admittance signatures of different damage extent; **(b)** RMSD value of different damage extent.

**Figure 11.**
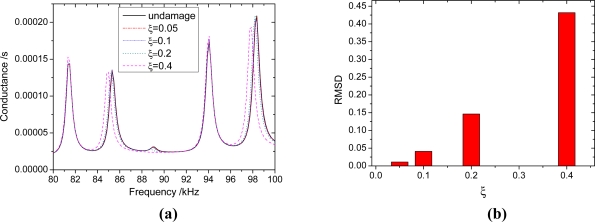
The influence of different extent crack damage in 80 kHz–100 kHz. **(a)** the admittance signatures of different damage extent; **(b)** RMSD value of different damage extent.

**Figure 12.**
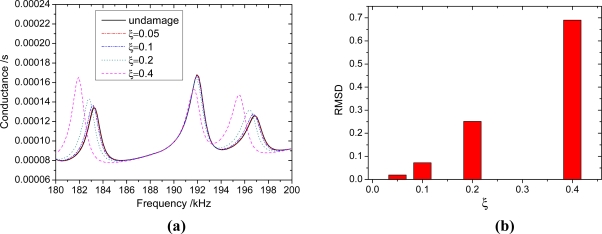
The influence of different extent crack damage in 180 kHz–200 kHz. **(a)** the admittance signatures of different damage extent; **(b)** RMSD value of different damage extent.

Consider a beam with a crack located at *x* = 100. The parameters of the beam are listed in Table 1, and the beam thickness *h_b_* = 5 mm. the depth of crack is *h_c_*, the relative depth *ξ* = *h_c_* / *h*_b_. To verify the reliability of the proposed method, the admittance signatures of PZT patch from Ansys are compared with the data obtained by the analytical model. The crack is modeled in the ANSYS 10.0 as a slot with a width of 0.1 mm. the size of mesh near the slot is smaller than that of other areas (Figure 8). The results are shown in Figure 9. From Figure 9, it can be seen that the proposed method can predict admittance signatures of damaged structural as well as the FEA method. In order to study the influence of the crack on the admittance signature of the PZT patch, the admittance signatures are calculated when *ξ* = 0.05, *ξ* = 0.1, *ξ* = 0.2 and *ξ* = 0.4, respectively. The corresponding results are shown in Figure 10, Figure 11 and Figure 12. The resonant peaks of the admittance signatures shift towards the left with increasing crack depth. The influence of the crack increased with increasing frequency. These results are in good accord with the experimental phenomena in reference [30] and reference [33]. In fact, the change of admittance signature and the decrease of the peek frequencies reflect the decreasing local stiffness due to the crack, so the EMI technique can be used to identify the crack damage. From the changes of the admittance signature caused by the appearance of damage, both the location and quantity of the damage can be identified by using a certain back-calculation algorithm.

The crack has little influence on the admittance signature in some frequency bands, such as 45 kHz–47 kHz in Figure 10, 80 kHz–83 kHz in Figure 11 and 191 kHz–193 kHz in Figure 12. The frequency band is related to the crack location. The same phenomenon was also found by Youdi [33]. The reason is that if the crack is located at the node of a displacement modal, the crack has no influence on the modal frequency; otherwise, the modal frequency will reduce with the increasing crack depth. Hence, in order to improve the damage identification accuracy, the sweeping frequency band needs to contain enough peek frequencies to avoid the error due to the absence of influence of the crack around some frequencies. From the signatures of different damage extent, we can calculate the RMSD (root mean square deviation) value as follows:

(35) $$\text{RMSD}=\sqrt{\frac{\sum_{i=1}^{n}{\left(Y_{i}^{d}-Y_{i}^{u}\right)}^{2}}{\sum_{i=1}^{n}{\left(Y_{i}^{u}\right)}^{2}}}$$

where *Y* is the admittance of PZT, the superscripts *d* and *u* denote the signature of the damaged structure and undamaged structure, respectively, and *n* denotes the number of sample points.

The RMSD values are shown in Figure 10, Figure 11 and Figure 12. From these figures, it can be seen that for the same extent of damage, the RMSD value increases with increasing frequencies. In other words, a higher frequency is more sensitive to damage than a lower frequency. However, when the driven frequency is higher than 200 kHz, the signature can be easily affected by temperature and bonding layer, hence, the driving frequencies used in EMI techniques are normally less than 200 kHz [35]. According to the engineering experience with the EMI technique, the minimum RMSD value which can be viewed as a reliable indicator of the existence of structural damage can be set as 1% [36]. In this work, the crack damage with a depth *h_c_* = 0.5 mm (*ξ* = 0.1) can be detected, as the RMSD value in the frequency range 180 kHz–200 kHz is greater than 5%.

## 5. Conclusions

An EMI model of a cracked Timoshenko beam with a pair of PZT patches system has been developed by considering the inertial forces of both the PZT patches and the bonding layers. The theoretical analysis and numerical tests are focused on the influence of crack and the influence of inertial forces because of the beam transverse motion. Through numerical tests results, the following conclusions can be drawn:

1. The inertial forces of PZT patches and bonding layers produced by the transverse motion of beam can be ignored in the low frequency band but should be considered in the high frequency band, especially for a thin beam structure. Because the EMI technique employs high frequency, taking the inertial forces into account is necessary when monitoring a thin beam structure.
2. The admittance signature of the PZT patch can reflect the crack damage very well, especially in the high frequency band. In some frequency bands, the crack has little influence on the admittance signature, while in other frequency bands the peak frequency of the admittance signature decreases with increasing frequency. In order to improve the accuracy of damage identification, the high frequency band which contains many peak frequencies should be chosen.

Based on the proposed EMI model, future work is planned to identify the crack damage quantitatively. To quantify the crack damage in plates and shells, further research is needed to establish the corresponding EMI models for cracked plates and cracked shells.

## Figures and Tables

**Table 1.** Properties of the beam.

*l*_b_**(mm)**	*b*_b_**(mm)**	*E*_b_**(Pa)**	*G*_b_**(Pa)**	**Poisson’s ratio**	*ρ*_b_**(kg/m^3^)**	**Damping ratio**
300	8	6.60E10	2.33E10	0.33	2700	0.01

**Table 2.** Properties of the PZT patch.

*l*_p_**(mm)**	*h*_p_**(mm)**	*b*_p_**(mm)**	*E*_p_**(Pa)**	*ρ*_p_**(kg/m^3^)**	*d*_31_**(m/V)**	*ε*_33_**(F/m)**	*δ*	*η*
10	0.5	8	6.1E10	7750	−1.71E-10	1.53E-8	0.02	0.03

**Table 3.** Properties of the adhesive layer.

*l*_a_**(mm)**	*h*_a_**(mm)**	*b*_a_**(mm)**	*G*_a_**(Pa)**	*ρ*_a_**(kg/m^3^)**	*C*_b_	**Poisson’s ratio**
10	0.1	8	1.0E9	1700	1.0E-7	0.38

**Table 4.** *γ vs. h*_b_.

*h*_b_**(mm)**	**2.5**	**5**	**10**	**30**
*γ*	3.321	2.116	1.580	1.193

**Table 5.** Constitutive data of the PZT-5A.

***S*(m^2^/N)**	***d*(C/N)**	***ε* (F/m)**
*S*_11_ = *S*_22_ = 16.4E-12	*d*_31_ = −171E-12	*ε*_11_ = 1.53E-8
*S*_12_ = −5.74E-12	*d*_32_ = −171E-12	*ε*_22_ = 1.53E-8
*S*_13_ = *S*_23_ = −7.2E-12	*d*_33_ = 374E-12	*ε*_33_ = 1.50E-8
*S*_33_ = 18.8E-12	*d*_42_ = 584E-12	
*S*_44_ = *S*_55_ = 47.5E-12	*d*_52_ = 584E-12	
*S*_66_ = 44.3E-12		
